# Space Power in Inclusive Development: Industrial Clusters and Rural Anti-Poverty

**DOI:** 10.3390/ijerph182010943

**Published:** 2021-10-18

**Authors:** Junqian Wu, Xiaoqian Liu, Jianqing Ruan, Xiulin Qi, Chang’an Wang, Dan Fan

**Affiliations:** 1 China Western Economic Research Center, Southwestern University of Finance and Economics, Chengdu 610074, China; wujunqian@swufe.edu.cn (J.W.); fandan@swufe.edu.cn (D.F.); 2 Research Institute of Economics and Management, Southwestern University of Finance and Economics, Chengdu 610074, China; wptaq11231@126.com (X.L.); wangtcjy@163.com (C.W.); 3 School of Public Affairs, Zhejiang University, Hangzhou 310058, China; 11020033@zju.edu.cn; 4 Business School, Zhengzhou University, Zhengzhou 450001, China

**Keywords:** inclusive development, industrial clusters, farmer’s income, anti-poverty

## Abstract

Poverty seriously hinders the inclusive development of mankind and is closely related to economic growth, ecological protection, ecological restoration and sustainable use of resources. Based on the data of economic census and rural fixed observation point, a spatial econometric model is established to test the direct impact and spatial spillover effect of industrial clusters on rural poverty alleviation. The result of household-level is that the number of industrial clusters has a negative effect on poverty, namely the farmers who live in the county with more industrial clusters, may be less likely to become the poor. The number of industrial clusters in other regions also has a negative effect on poverty. By dividing farmers into the poverty and non-poverty group, the study finds that, for the poverty group, the number of industrial clusters has a positive direct and spillover effect on farmers’ income. For the non-poverty group, the number of local industrial clusters has a positive direct effect on farmers’ income, but the number of industrial clusters in other regions does not have any effects or has a negative direct effect on farmers’ income. By classifying the industries, the study discovers that the labor-intensive industrial clusters, such as textiles, manufacture and processing of machinery parts and paper industries, have a positive effect on farmers’ income.

## 1. Introduction

Poverty seriously hinders inclusive development, as well as ecological protection, ecological restoration and sustainable use of resources. Anti-poverty has always been a key issue of concern to the academic and policy circles. This article studies the space power in inclusive development. From the great geographical discovery to the industrial revolution, from the “migrant worker tide” to “the world is flat”, the constant struggle against the “hegemony of distance” and the pursuit of “collection of profits” constitute the history of the development of human society [1]. Traditional research suggests that economic agglomeration can increase wages or labor productivity and thus reduce poverty. However, the impact of economic agglomeration on poverty reduction for farmers is uncertain. Economic agglomeration increases the wage income of farmers because of the “scale effect”, while it may also cause the loss of agricultural production resources due to the “siphon effect” and in turn reduce farmers’ agricultural income. Therefore, the impact of economic agglomeration on poverty alleviation in rural areas is still an undeveloped theme. In addition, as traffic conditions have been improving and institutional barriers are gradually eliminated, the links between regions are increasingly closer, so the “spatial spillover effect” caused by economic agglomeration must be considered. If we only study the impact of local economic agglomeration on rural poverty reduction, we may miscalculate that poverty reduction effect. Can economic agglomeration really benefit farmers and reduce rural poverty?

In practice, under the dual disadvantages of natural resource endowments and national policies, aggregate economic output and farmers’ income of Zhejiang province has grown rapidly, the success of which lies in the development model of industrial clusters [2]. This model has practically verified the effect of economic agglomeration on economic growth. Industrial clusters, the important form of economic agglomeration, can better target poor farmers than generalized economic agglomeration. Moreover, because the enterprises in the industrial clusters are mainly small and medium-sized labor-intensive enterprises, they can bring in a lot of job opportunities. For the poor farmers, their largest capital is the labor force. Once they have the chance of engaging in non-agricultural work, they can immediately mitigate poverty [2]. Therefore, we may mainly focus on industrial clusters of Zhejiang province to study their impacts on the increase of farmers’ income and poverty alleviation.

The role of industrial clusters in rural anti-poverty is on the basis of many important theories. The new economic geography theory believes that industrial clusters can promote economic growth and reduce poverty through external ties. Specifically, economic agglomeration may have an effect on revenue growth through three micro-mechanisms—sharing, matching and learning [3]. Based on the decision theory, in addition to the above three mechanisms, changes in farmers’ identities and the environment will also increase their income and reduce rural poverty [4]. Nevertheless, new economic geography theory seeks the balanced relationship between scale benefits brought by economic agglomeration and transport cost caused by distance [5]. It is believed that economic agglomeration in a region, can only bring about the welfare for the local farmers and the distance creates a kind of “tyranny of distance” that prevents farmers who are far from the agglomeration region from enjoying such benefits. Spatial econometric explains the meaning of the distance from a different perspective. Different from the traditional econometric hypothesis that the samples between each other are independent, spatial econometric suppose samples have spatial correlations. It puts spatial distance weights into the spatial econometric model and reasonably constructs the effect of economic agglomeration in other regions on the growth of local farmers’ income and poverty alleviation, all of which make the distance no longer a “distance autocracy” but a link between the regions. Therefore, this paper not only stresses the effect of local industrial clusters on increasing farmers’ income and rural anti-poverty but also uses spatial econometric models to mainly focus on the role of industrial clusters in other regions in the increase of local farmers’ income and rural poverty alleviation.

## 2. Literature Review

According to the analytical framework of Nadvi and Barrientos [6], the effects of industrial clusters on poverty reduction include direct and indirect effects. The direct effect is that farmers can participate in the cluster, which includes three aspects. The first is farmers can receive job opportunities. McCormick [7] analyzes six cases of industrial clusters in Africa and finds that although industrial clusters only provide low-paid job opportunities, these job opportunities can help families survive and escape from poverty. Through investigating the evolution of industrial clusters of children’s clothing in Zhili County, Zhejiang Province, China, Fleisher et al. [8] argue that these industrial clusters have employed more than 200,000 workers by 2005, accounting for approximately one-third of the employment of this industry in China. According to statistics on industrial clusters in Wenzhou City, Zhejiang Province, Sonobe et al. [9] draw a conclusion that the average number of employees in Wenzhou increases from 47 in 1990 to 339 in 2000. The second is farmers’ income can be increased. Many studies conclude that industrial clusters can raise workers’ wages. Sonobe et al. [9] emphasize industrial clusters have played an important role in increasing labor productivity. Visser [10] examines Peru’s garment, he finds that the wage of firms in the cluster is higher than that of firms not in the cluster in the same industry. Fowler and Kleit [11] consider that the number of industrial clusters has a significant effect on reducing poverty. The third is to enhance the ability. The feasible competence theory proposed by Amartya Sen indicates that poverty deprives the basic ability or power, not only low incomes [12]. Because technology and information may spill in a region, the enterprises in the industrial clusters can further promote the spread and application of new knowledge, technologies and ideas among enterprises. Through “learning by doing” in the cluster, the capabilities of skills and management of entrepreneurs have been improved [9]. In addition, industrial clusters also allow the poor to have more rights, especially for women, which is also an indication of improving their ability. Batra and Aneja [13] explore the role of UNIDO’s textile cluster in Chanderi in improving women’s textile capacity, increasing their self-esteem and involving women more in industrial clusters; the indirect effect is mainly reflected in the externalities of the industrial clusters. First, agglomeration economies and joint actions. Agglomeration economies can reduce costs and allow small businesses to enter the market, which can increase their chances of obtaining jobs and improve their abilities to obtain more income. Cluster joint actions can further strengthen the capacity of local firms and reduce the vulnerability to external shocks [6]. Second, in terms of promoting local economic development: Promoting the development of industrial clusters is widely regarded as a valuable goal of regional economic development [11]. Industrial clusters, through providing high-quality products and services, can improve infrastructure, which further attracts suppliers, customers and public investors to invest in the infrastructure, which may benefit the whole business development in this region [14].

There are few empirical articles on the role of industrial clusters in poverty alleviation. Nadvi and Barrientos [6] believe that the role of industrial clusters in poverty alleviation is a subject that remains to be developed. They analyze this subject from three perspectives: The first is cluster characteristics. Some types of clusters may directly affect poverty, such as rural clusters, informal urban clusters, small and medium enterprises clusters, micro-enterprise or family workshop clusters, labor-intensive industrial clusters and employees consisting of women, migrant workers or non-skilled workers. The second is the processing of clusters. The scale effect of economic agglomeration can reduce transaction costs and improve employee capabilities. The third is cluster dynamics. Through joint operations, the capabilities of local companies have been improved and the risks caused by external shocks have been reduced. Fowler and Kleit [11] are the first and probably the only ones to systematically verify the relationship between industrial clusters and poverty. Based on cross-sectional data at the county level in the United States, they used the spatial error model, under the control of the population, local economic factors, local geographical and spatial factors, concluding that the number and depth of industrial clusters in small and medium-sized cities across the country have a positive and significant effect on the reduction of poverty, while in big cities, the number of industrial clusters does not significantly reduce poverty. Long and Zhang [15], although not directly studying the relationship between industrial clusters and poverty, focus on the research of the performance of industrial clusters, which is less involved in previous studies. Through the employ of China’s industrial census in 1995 and the economic census of enterprise-level micro-data in 2004, they emphasize that firms have better and full factor growth in some regions where China’s industrial clusters have developed well.

Industrial clusters may affect poverty in a variety of ways. The affection channels are mainly through farmers participate in industrial clusters, to obtain more job opportunities and non-agricultural income; through the externalities of industrial clusters, local farmers can obtain more convenient infrastructure and obtain access to the dripping effect from regional economic development. Based on this, Fowler and Kleit [11] have added that due to population migration, the population base in the poverty regions will be reduced or increased, which in turn may increase or decrease the incidence of poverty. They provide a good theoretical stance and empirical reference for the study of the role of industrial clusters in poverty. However, industrial clusters in the United States are the agglomeration of large companies, while in China are the agglomeration of SMEs. The differences are relatively large. More importantly, the economic society environment, resource endowment and poverty conditions in the United States and China are also quite different. What is the role of industrial clusters with Chinese characteristics in poverty alleviation? Therefore, from the household-level, this paper will examine the impact of the number of industrial clusters, the type of industrial agglomerations and agglomeration levels on farmers’ income and rural poverty reduction.

## 3. The Conceptual Model

Industrial clusters may influence poverty reduction in rural areas through external ties. Microcosmic mechanisms include sharing, matching, learning, identity and environment [3]. Firstly, sharing: industrial clusters enable companies to effectively share inseparable services or facilities, intermediate inputs, external risks, specialization and diversification [6,16,17,18,19,20]. Secondly, matching: industrial clusters can better match workers and companies, companies in related industries, sellers and buyers, entrepreneurs and capital markets [3,17,19]. Thirdly, learning: workers acquire knowledge through “learning by doing” and then improve human capital; face-to-face exchanges brought by spatial agglomeration of a large number of economic activity entities can promote the exchange and dissemination of knowledge and technology; continuous exchanging and learning promote the accumulation of knowledge, which, in turn, promotes the creation of new knowledge and new ideas. These processes can all in different degrees improve the efficiency of production [3,21].

For farmers, the impact of industrial clusters on farmers’ income is also reflected in the following three aspects. First, the status changes. Through entering the environment of industrial clusters, farmers have gained non-agricultural employment opportunities and non-agricultural income. Second, changes in the non-agricultural employment environment. Farmers can obtain non-agricultural income not only through the local employment environment generated by industrial clusters but also because of spatial spillover effects, farmers can choose employment opportunities in different regions. Third, the changes of the agricultural environment. Industrial clusters have promoted the development of urbanization; of course, it is undeniable that industrial clusters also absorb the advantages of rural resources, which may result in the reduction of farmers’ agricultural income. Fourth, the changes of the living environment. The living environment emphasizes that irrespective of whether farmers have gained employment opportunities in industrial clusters, due to the existence of the price index effect, farmers can enjoy a lower consumer price index owing to industrial clusters, and thereby can increase real wages. In addition, the improvement of the infrastructure brought by the industrial clusters has made the life of the farmers more convenient, which to a certain extent has affected the income of the farmers; the industrial clusters have promoted the development of the local economy and enabled dripping effect of the economy to benefit farmers [1].

**Figure 1:**
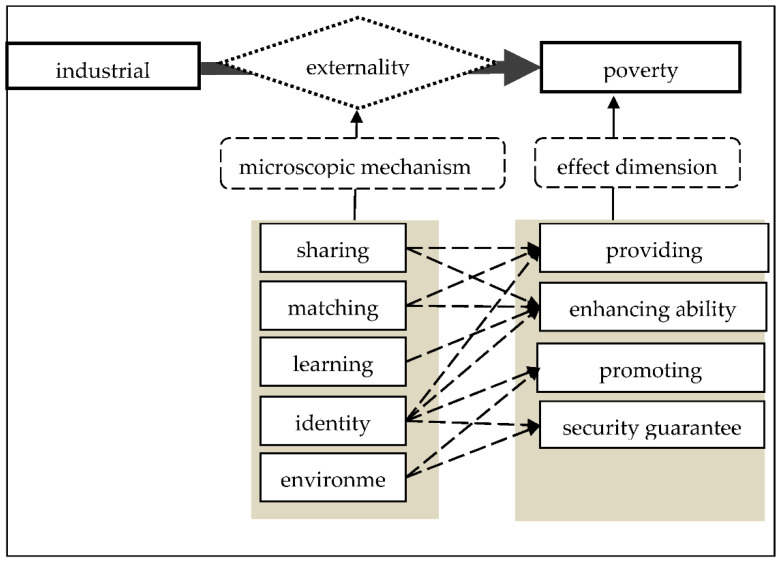
The conceptual model framework.

In response to the reduction of farmers’ poverty, based on the framework of the World Bank [22], this paper further strengthens the role of industrial clusters from four aspects—providing opportunities, enhancing capabilities, promoting empowerment and strengthening safety and security (Figure 1). Among them, industrial clusters provide farmers with low thresholds of work through sharing and matching mechanisms, enabling poor farmers to gain employment opportunities and improve work skills. Through the learning mechanism, poor farmers have enhanced their employment ability in the process of “learning by doing” or knowledge spillovers. Through the acquisition of identities, poor farmers have obtained employment opportunities and have enhanced their capabilities. After the poor farmers receive the status of workers, they can enjoy certain benefits such as labor insurance and legal protection provided by the enterprises, such as five insurances and public housing funds. Furthermore, they can obtain corresponding employment, life and political rights and reduce risks, such as poverty caused by illnesses and unemployment and receive security guarantees to some extent. The environment of the industrial clusters in which poor farmers are located also provides them certain rights and security guarantees, such as improvements in infrastructure, living conditions and social security. Moreover, whether farmers obtain employment opportunities or obtain the identity of workers in the environment or not, the farmers in the environment can enjoy the benefits. Based on the above analysis, we obtain two hypotheses:

**Hypothesis 1 (H1).**  *Industrial clusters can reduce the level of local rural poverty.*

**Hypothesis 2 (H2).**  *Industrial clusters can reduce the level of rural poverty in other regions.*

## 4. Research Design

### 4.1. Data Source

The data for calculating the relevant indexes of industrial clusters comes from the first and second economic the census in Zhejiang Province, the investigation time was 31 December 2004 and 31 December 2008. All legal entities engaged in the secondary and tertiary industries, industrial activity units and self-employed households were all the census objects. The content of census contains attributes of the unit, employees, financial status, production capacity, production, operation status, raw materials and energy consumption and scientific and technological activities. The first economic census conducted a random inspection of the data quality of 11 cities across the province. A total of 12,026 corporate units and industrial activity units in 126 census communities (with a sampling rate of 24.1‰) and 34,786 self-employed households (with a sampling rate of 12.6‰) were sampled. The spot-check summary results show that the overall error rates for the data collected from the first and the second economic census in Zhejiang province are 3.7‰ and 3.6‰. The error rate of the self-employed households is 1.8‰. The quality of the two economic census data all reached the expected target (the second census leadership group office of the People Government of Zhejiang Province, 2005; 2010). The use of economic census data is due to the fact that macro-statistical data or Chinese industrial enterprise databases used in previous studies often only publish data of enterprises above designated size, while the characteristics of Chinese industrial clusters are characterized by small and medium-sized enterprises. Therefore, economic census data used to study industrial clusters may be more accurate and scientific.

The data of economic census were used to match the micro-level data from household-level coming from the rural fixed observation point of Zhejiang province in 2004 and 2008, and the data is a follow-up survey data of 500 households of 10 villages in Zhejiang province. It is an official and authoritative large-scale micro survey data that can accurately reflect the development of agriculture, rural areas and farmers. Due to the use of a fixed household survey, the data in this paper can form panel data.

### 4.2. Econometric Model

As a developed province of China, Zhejiang province has become China’s first province to eliminate “poverty counties” in 1997 and the poverty line has been greatly improved. Therefore, this paper will use 50% of the average income of all farmers as the poverty line. For comparison purposes, the poverty line in 2004 and 2008 will remain unchanged. The poverty line was defined by the Organization for Economic Cooperation and Development (OECD) in 1976 and has become a widely used poverty standard. On this basis, this paper uses the panel binary logistic model to estimate parameters.

Because the explanatory variable in this paper is a 0–1 variable, we choose the logit model for regression. Compared with the linear regression model, the logit model can effectively overcome the problems of heteroskedasticity and estimation bias. The logical probability distribution function is as follows:

(1) $$P_{i}=F(Y_{i})=F(\alpha +\beta X_{i})=\frac{1}{1+\exp^{-Y_{i}}}=\frac{1}{1+\exp^{-(\alpha +\beta X_{i})}}$$

where exp represents the base of the natural logarithm, $Y_{i}=\alpha +\beta X_{i}$, *α* is the intercept term; *β* is a vector of regression coefficients to be estimated and *X* is a set of independent variables determining the probability of the event. For a given *X_i_*, *P_i_* is the probability of the event.

This paper sets the dependent variable as the probability of poverty, which is a binary dependent variable. *Y* equals 1 when a farmer is poor and otherwise *Y* equals 0; if *y* = 1, the probability is *P*, then the distribution function of *y* is:

(2) $$f(y)=p^{y}{\left(1-p\right)}^{1-y};y=0,1$$

Therefore, from the above two equations, we can conclude that the regression function is:

(3) $$\mathrm{logit}p=\ln\frac{p}{1-p}=\alpha +\sum_{i=1}^{n}\beta_{i}x_{i}$$

Among them, $\frac{p}{1-p}$ is the relative risk (or odds) and represents the probability of an occurrence of the event relative to the likelihood of a nonoccurrence given *X*, *β_i_* is a vector of regression coefficients to be estimated. If *p* > 0.5, this event can be predicted to happen, otherwise this event does not occur. The econometric model is as follows:

(4) $$\begin{array}{l} \mathrm{logit}(poverty_{it})=\alpha_{0}+\beta_{1}cluster_{it}+\beta_{2}\sum_{j=1}^{N}w_{ij}cluster_{jt}+\beta_{3}age_{it}+\beta_{4}edu_{it}\\ +\beta_{5}pop_{it}+\beta_{6}land_{it}+\beta_{7}capital_{it}+\mu \end{array}$$

The independent variable $\overset{N}{\underset{j=1}{\sum }}w_{ij}cluster_{jt}$ represents the sum of the number of local industrial clusters multiplied by the reciprocal of the economic distance. The economic distance is measured by the actual traffic time between the two counties. The traffic time is based on the geographical distribution of the counties in Zhejiang province. The google map is used to check the time spent driving motor vehicles between administrative centers of the two places. The economic distance is measured by the actual traffic time between the two counties. The traffic time is based on the geographical distribution of the counties in Zhejiang province. The google map is used to check the time spent driving motor vehicles between administrative centers of the two places.

The control variables are respectively the age and the education level (edu) of the householder, the number of family members (pop), the land owned by the family (land) and the family capital (capital).

Model 1 and model 2 use the binary logistic model. The dependent variable is the probability of poverty. If farmers’ income is less than the poverty line, Y equals 1 and otherwise Y equals 0 (Table 1). Model 1 employs 50% of the average income of the farmers in 2004, i.e., 10,234 China yuan, as the poverty line. Model 2 uses 50% of the average income of farmers in 2008, i.e., 12,042.48 yuan, as the poverty line. To ensure the robustness of the model, Model 3 uses the fixed-effect model, and the dependent variable is the natural logarithm of farmers’ per capita net income.

## 5. Results

### 5.1. Preliminary Estimation Results

It can be seen that the result in Model 1 is consistent with that in Model 2 and Model 3. The number of local industrial clusters has a negative and significant effect on the probability of poverty. That is, the more industrial clusters in counties, farmers in these counties are less likely to become the poor. This verifies Hypothesis 1. The number of industrial clusters in other regions has a negative impact on the probability of poverty. This verifies Hypothesis 2. The number of local industrial clusters and industrial clusters in other regions both has a positive and significant impact on the growth of farmers’ income.

### 5.2. Robustness Test and GMM Model Estimation Results

Although the number of industrial clusters is the most intuitive and simplest indicator to measure the development level of industrial clusters, the estimated results are real and effective. In order to make this conclusion more cautious, this paper employs economic density (the total GDP per unit area) as an alternative to the number of industrial clusters, further estimations results are in Model 4 and Model 5 (Table 2). The dependent variable of Model 4 is farmers’ per capita net income and the dependent variable of Model 5 is the probability of poverty. The estimated results for Model 4 and Model 5 are consistent with the previous models, showing that the models are robust.

The number of industrial clusters, the main explanatory variable, may be endogenous. On one hand, the number of industrial clusters may increase farmers’ income; on the other hand, industrial clusters may also be influenced by the increase of farmers’ income. Farmers engage in non-agricultural industries and then they may become entrepreneurs, so the large concentration of enterprises may in turn create new industrial clusters. Therefore, we use the system GMM panel model to estimate the parameters and overcome the endogenous problem of each explanatory variable in the model.

This paper employs the county’s GDP of a decade ago as an instrumental variable because the generation of industrial clusters is a process of historical evolution and accumulation. The regional GDP of a decade ago may have an effect on the industrial conditions and the number of enterprises at that time, and therefore it is related to the number of existing industrial clusters; ten-year intervals is long enough, so the regional GDP of a decade ago is almost irrelevant to the current micro farmers’ income. The dependent variables were the farmers’ income and the probability of poverty, the system GMM panel model is used to estimate the regression. The results of Model 6 and Model 7 are the same as those of Model 1 to Model 5, described above. It can be seen that the results are robust, and the endogenous biases are effectively eliminated (Table 3).

### 5.3. Estimated Results of Differentiating Farmers

It is worth noting that from the perspective of the employment structure in the actual industrial clusters, local farmers can be divided into two categories. The first category is that farmers have stable jobs in industrial clusters and they may already be entrepreneurs, who have a high level of expertise, strong asset specificity and low liquidity. Industrial clusters in other regions have a weak influence on them. The second category of farmers assets has low specificity, while workflow and geographical mobility are very strong. Local industrial clusters and industrial clusters in other regions both have impacts on them. These impacts are not because of the specialized production of industrial clusters but come from low threshold employment opportunities arising from economic agglomeration.

Therefore, in order to verify this conclusion, this paper classifies farmers into poverty and non-poverty groups according to farmers’ income. Specifically, according to the 2004 relative poverty line, farmers below this standard are classified as poverty group and farmers above this standard are classified as the non-poverty group. The system generalized momentum panel model is used to estimate in Model 8 and Model 9. Model 8 is an estimate of the poverty group. Model 9 is an estimate of the non-poverty group (Table 4).

From the estimation results, it can be seen that for the poverty-stricken farmers, the number of local industrial clusters and the number of industrial clusters in other regions both have positive effects on the farmers’ income. Farmers with lower income are the ones with low asset specificity and have very strong liquidity and geographic mobility. The professional division of labor within the industrial clusters and the labor-intensive characteristics provide this kind of farmers with a large number of low-threshold employment opportunities. Moreover, even if farmers do not obtain jobs directly in industrial clusters, farmers can still benefit from the externalities brought by industrial clusters; for the non-poverty group, their assets are highly specific and “locked-in” in the local industrial clusters; besides, because industrial clusters in other regions compete with local industrial clusters, this also has a negative impact on their income.

### 5.4. Estimated Results of Classifying Industries

Different types of clusters may have different effects on poverty [6]. Fowler and Kleit [11] conclude that industrial clusters in different industries have different impacts on poverty. Therefore, in order to further explore what types of clusters have a greater impact on farmers’ income, this section will examine the impact of clusters in different industries on farmers’ income.

The three digit and four digit industry codes are used to calculate the number of industrial clusters in different counties, and the data are merged with the rural household micro-data coming from rural fixed observation points. The corresponding industries in 2004 and 2008 are different, so they are divided into two cross-sectional data. Through the measurement, the estimated results in 2004 and 2008 are respectively shown in Table 5 and Table 6. In order to intuitively analyze the results, this paper sorts out the significant industries corresponding to industrial clusters, which are all in line with the theoretical expectations. Judging from the results of classifying industries in 2004, the significant industries mainly are industries such as textile and manufacture and processing of mechanical parts. Judging from the results of classifying industries in 2008, the significant industries are mainly related industries such as paper industries, textiles and corporate management. It is obviously showed that visible industries are all labor-intensive.

## 6. Conclusions

Through the regression analysis from the household level, this paper concludes that the number of local industrial clusters has a positive and significant impact on the growth of farmers’ income, and has a negative and significant impact on the probability of poverty. Namely the farmers who live in the county with more industrial clusters, may be less likely to become the poor. The number of industrial clusters in other regions has a positive impact on farmers’ income and has a negative and significant effect on the probability of poverty. By dividing farmers into the poverty and non-poverty group, it is found that for the poverty group, farmers’ asset specificity is low while their work mobility and geographical mobility are strong. The number of local industrial clusters and the number of industrial clusters in other regions both have positive effects on the farmers’ income. Because of the professional division of labor within the industrial clusters and the labor-intensive characteristics, these farmers are provided with a large number of low-threshold employment opportunities. Even if farmers do not obtain jobs directly in industrial clusters, farmers can still benefit from the externalities brought by industrial clusters; for the non-poverty group, they have a high level of professional skills and strong asset specificity but weak work and regional mobility. Therefore, the number of local industrial clusters has a positive impact on their income. As industrial clusters in other regions compete with local industrial clusters, industrial clusters in other regions have a negative impact on their income. According to the analysis of classifying industries, the types of industries that industrial clusters have a significant impact on farmers’ income and rural poverty reduction are mainly labor-intensive industries such as textiles, manufacture and processing of machinery parts and paper industries.

Industrial clusters have a direct and spatial spillover effect on poverty alleviation. This policy inspires us to fully utilize the multi-function of industrial clusters and establishes a cluster-based anti-poverty model. Give full play to the pro-poor functions of industrial clusters. Industrial clusters can serve as pro-poor institutions [6]. Under the backdrop of inclusive growth, we should emphasize the friendly development of the environment and resources of industrial clusters, actively promoting the benefits of industrial clusters to be enjoyed by the poor farmers and narrow the poverty gap and the gap between urban and rural areas. They can also play the targeting function of industrial clusters. At present, the two directions of anti-poverty are targets and the dripping effect of economic growth. Industrial clusters are a trade-off between the two. Because the characteristics of industrial clusters can absorb a large number of poor farmers, the industrial clusters themselves are a kind of “self-target”, which can reduce the E-error and F-error of the target. Moreover, industrial clusters can bring companies together and make policies more effective [6]. Therefore, adopting a development policy aimed at industrial clusters can make the object of policy clearer and strengthen the role of policies in increasing employment and income. Play the community functions of industrial clusters. Hayami and Godo [23] define the community as a group of closely interacting people who are linked to one another through mutual trust, who believe that economic development needs to organically combine the market, country and community together and emphasize the reduction effect of the community on the probability of poverty.

Compared with the market, the cluster has its independence; compared with the enterprise, the cluster has its stability. Because of the close correlation and competition among the enterprises in the cluster, the enterprise’s dishonest behavior is reduced. Therefore, industrial clusters that are closely linked with social capital can fully play the role of the community and strengthen the government’s support for the infrastructure, public services and social security, so that people within the industrial clusters can have fair chances. They can promote their capabilities to improve, give them more life and political rights and build a safety net for them.

Although the data used for the empirical study in this paper are mainly from mainland China, the conclusions of this paper are somewhat generalizable. The industrial cluster-based development model was successful not only in Italy in the late 1970s, but also other East Asian countries and Africa [2]. Industrial clusters provided the impetus for inclusive development in these regions. However, there are still some limitations of this paper: First, this paper examines the impact of industrial clusters on rural anti-poverty in a local region of mainland China, but in today’s increasingly globalized world, there may be implications between industrial clusters and inclusive development in different countries and different regions. Second, there is no conclusive scientific definition of industrial clusters. While this paper has attempted to develop some measures in our study, a more accurate measure of industrial clusters would help to enhance the robustness of our research findings. Third, industrial clusters may bring about problems such as environmental pollution while promoting rural anti-poverty, and how to measure the impact of industrial clusters comprehensively is also a direction for our further research.

## Figures and Tables

**Table 1:** Estimates of industrial clusters and poverty reduction.

Dependent Variable	The Probability of Poverty (2004)	The Probability of Poverty (2008)	ln(Farmers’ per Capita Net Income)
	Logit	Logit	Fe
	Model 1	Model 2	Model 3
local industrial clusters	−0.01 **	−0.01 ***	0.01 **
	(−2.00)	(−2.61)	(2.32)
industrial clusters in other regions	−0.25 ***	−0.24 ***	0.16 ***
	(−3.96)	(−4.61)	(4.97)
age	0.02 **	0.02 **	−0.005
	(2.41)	(2.18)	(−0.96)
education	−0.04	−0.05 *	0.05 ***
	(−1.44)	(−1.66)	(3.19)
gender	0.06	0.11	−0.12
	(0.18)	(0.35)	(−0.64)
pop	0.25 ***	0.24 ***	−0.12 ***
	(3.62)	(3.76)	(−3.33)
land	0.11 **	0.09 **	−0.04
	(2.35)	(2.13)	(−1.39)
capital	−3.7 ***	−3.5 ***	2.9 ***
	(−5.38)	(−6.01)	(8.97)
intercept item	−0.72	−0.23	9.17 ***
	(−0.92)	(−0.31)	(21.22)
observations	975	975	974

Note: The reported result is the regression coefficient and the value in the bracket is t. * Indicates that the 10% level is significant; ** indicates that the 5% level is significant; *** indicates that the 1% level is significant.

**Table 2:** Robustness test of economic density on farmers’ income.

Dependent Variable	ln(Farmers’ per Capita Net Income)	the Probability of Poverty (2004)
	Model 4	Model 5
economic density	1.95 ***	−1.18 ***
	(5.95)	(−3.10)
age	−0.003	0.02 *
	(−0.62)	(1.87)
education	0.05 ***	−0.04
	(3.17)	(−1.41)
gender	−0.12	0.02
	(−0.61)	(0.07)
pop	−0.11 ***	0.22 ***
	(−3.00)	(3.39)
land	−0.03	0.12 ***
	(−1.28)	(2.69)
capital	3.01 ***	−3.43 ***
	(9.31)	(−5.24)
intercept item	9.42 ***	−1.31 *
	(21.98)	(−1.82)
observations	974	975

Note: The reported result is the regression coefficient and the value in the bracket is t. * Indicates that the 10% level is significant; ** indicates that the 5% level is significant; *** indicates that the 1% level is significant.

**Table 3:** The estimation results of the GMM model.

Dependent Variable	ln(Farmers’ per Capita Net Income)	The Probability of Poverty (2004)
	Model 6	Model 7
local industrial clusters	0.004 ***	−0.001 *
	(2.88)	(−1.88)
industrial clusters in other regions	0.07 ***	−0.04 ***
	(3.26)	(−3.46)
age	−0.01 *	0.004 **
	(−1.78)	(2.47)
education	0.03 ***	−0.01 *
	(2.81)	(−1.90)
gender	−0.23 *	0.02
	(−1.66)	(0.34)
pop	−0.12 ***	0.04 ***
	(−4.46)	(2.94)
land	−0.05 ***	0.02 **
	(−2.59)	(2.19)
capital	2.71 ***	−5.52 ***
	(11.77)	(−4.91)
intercept item	9.98 ***	0.27 *
	(31.92)	(1.75)
observations	974	975

Note: The reported result is the regression coefficient and the value in the bracket is t. * Indicates that the 10% level is significant; ** indicates that the 5% level is significant; *** indicates that the 1% level is significant.

**Table 4:** Estimates of poverty and non-poverty.

Dependent Variable	ln(Farmers’ Per Capita Net Income)	
	The Poverty	Non-Poverty
	Model 8	Model 9
local industrial clusters	0.004 *	0.004 **
	(1.86)	(2.31)
industrial clusters in other regions	0.22 ***	−0.06 **
	(7.09)	(−2.39)
age	−0.01	−0.001
	(−1.21)	(−0.26)
education	0.03 **	0.04 **
	(2.03)	(2.50)
gender	−0.004	−0.33 *
	(−0.02)	(−1.93)
pop	−0.15 ***	−0.10 ***
	(−4.15)	(−2.98)
land	−0.03	−0.02
	(−1.28)	(−1.03)
capital	2.91 ***	1.95 ***
	(8.88)	(6.98)
intercept item	8.75 ***	10.74 ***
	(19.25)	(29.28)
observations	475	499

Note: The reported result is the regression coefficient and the value in the bracket is t. * Indicates that the 10% level is significant; ** indicates that the 5% level is significant; *** indicates that the 1% level is significant.

**Table 5:** The estimation results of classifying industries in 2004.

Dependent Variable	ln(Farmers’ Per Capita Net Income)		The Probability of Poverty (2004)	
Code	Industry Names	Results	Industry Names	Results
Three Digits	Spinning, weaving and finishing of textiles	0.66 ***	Dyeing and finishing of cotton and chemical fiber textiles	−3.96 ***
	Manufacture of parts for general-purpose machinery and mechanical repair	0.16 ***	Metal surface treatment and heat treatment	−3.30 ***
			Manufacture of parts for general-purpose machinery and mechanical repair	−2.76 ***
			Spinning, weaving and finishing of textiles	−1.15 **
Four Digits	Spinning and weaving of silk textiles	0.66 ***	Manufacture of wool knitwear	−4.51 ***
	Manufacture of paper and cardboard containers	0.31 ***	Metal surface treatment and heat treatment	−1.51 ***
	Fasteners, springs manufacturing	0.33 *	Manufacture of textile and garment	−1.15 **
			Inorganic salt manufacturing	−0.97 *

Note: The reported result is the regression coefficient and the value in the bracket is t. * indicates that the 10% level is significant; ** indicates that the 5% level is significant; *** indicates that the 1% level is significant.

**Table 6:** The estimation results of dividing industries in 2008.

Code	Dependent Variables			
	ln(Farmers’ Per Capita Net Income)		The Probability of Poverty (2004)	
Three Digits	Paper industries	−1.16 *	Wholesale of textile, clothing and daily necessities	0.19 ***
Four Digits	Manufacture of paper and paperboard making machinery	−1.16 *	Corporate production management Service	0.26 *

Note: The reported result is the regression coefficient and the value in the bracket is t. * Indicates that the 10% level is significant; ** indicates that the 5% level is significant; *** indicates that the 1% level is significant.

## Data Availability

The published statistical data comes from the website (http://tjj.zj.gov.cn/col/col1530862/index.html) and (http://tjj.zj.gov.cn/col/col1530855/index.html) (both accessed on 11 August 2019).
